# Computational Fluid Dynamics Analysis and Empirical Evaluation of Carboxymethylcellulose/Alginate 3D Bioprinting Inks for Screw-Based Microextrusion

**DOI:** 10.3390/polym16081137

**Published:** 2024-04-18

**Authors:** Sungmin Lee, Minjae Son, Juo Lee, Iksong Byun, Jin-Woo Kim, Jungsil Kim, Hoon Seonwoo

**Affiliations:** 1 Department of Human Harmonized Robotics, College of Engineering, Sunchon National University, Suncheon 57922, Republic of Korea; 2 Interdisciplinary Program in IT-Bio Convergence System, Sunchon National University, Suncheon 57922, Republic of Korea; 3 Department of Aerospace Engineering, Graduate School, College of Engineering, Sunchon National University, Suncheon 57922, Republic of Korea; 4 Department of Animal Science & Technology, College of Life Sciences and Natural Resources, Sunchon National University, Suncheon 57922, Republic of Korea; 5 Department of Agricultural Machinery Engineering, College of Life Sciences and Natural Resources, Sunchon National University, Suncheon 57922, Republic of Korea; 6 Department of Biological & Agricultural Engineering, University of Arkansas, Fayetteville, AR 72701, USA; 7 Materials Science & Engineering Program, University of Arkansas, Fayetteville, AR 72701, USA; 8 Department of Smart Bio-Industrial Mechanical Engineering, College of Agriculture and Life Sciences, Kyungpook National University, Daegu 41566, Republic of Korea; 9 Department of Convergent Biosystems Engineering, College of Life Science and Natural Resources, Sunchon National University, Suncheon 57922, Republic of Korea

**Keywords:** screw-based dispenser system (SDS), computational fluid dynamics (CFD), non-linear regression analysis, wall shear stress, bioprinting, cell viability

## Abstract

Three-dimensional microextrusion bioprinting technology uses pneumatics, pistons, or screws to transfer and extrude bioinks containing biomaterials and cells to print biological tissues and organs. Computational fluid dynamics (CFD) analysis can simulate the flow characteristics of bioinks in a control volume, and the effect on cell viability can be predicted by calculating the physical quantities. In this study, we developed an analysis system to predict the effect of a screw-based dispenser system (SDS) on cell viability in bioinks through rheological and CFD analyses. Furthermore, carboxymethylcellulose/alginate-based bioinks were used for the empirical evaluation of high-viscous bioinks. The viscosity of bioinks was determined by rheological measurement, and the viscosity coefficient for the CFD analysis was derived from a correlation equation by non-linear regression analysis. The mass flow rate derived from the analysis was successfully validated by comparison with that from the empirical evaluation. Finally, the cell viability was confirmed after bioprinting with bioinks containing C2C12 cells, suggesting that the developed SDS may be suitable for application in the field of bioengineering. Consequently, the developed bioink analysis system is applicable to a wide range of systems and materials, contributing to time and cost savings in the bioengineering industry.

## 1. Introduction

Bioprinting is a method used to create complex structures by layering three-dimensional (3D) biological structures using bioinks containing cells, biomaterials, growth factors, and biocompatible polymers [1,2], which is a promising technology because it can be used to fabricate drugs, medical implants [3,4], scaffolds [5,6], replacement cells [7,8], and organs [9,10]. The bioprinting methods use inkjet, ultraviolet (UV)-assisted, and microextrusion bioprinting [11,12]. Among them, microextrusion bioprinting is a method of extruding bioink through a nozzle by applying pressure to a syringe containing bioink, which can produce complex tissue structures with high resolution and high cell viability by controlling printing parameters such as pressure, nozzle size, and printing speed [13,14]. Microextrusion bioprinting is the most popular of the bioprinting methods and is the primary method for printing cell-containing bioinks [15,16]. Types of microextrusion bioprinting include pneumatic pump, piston, and screw methods [17,18]. Among them, the screw-based method is capable of printing low to high-viscous bioinks. In particular, high-viscous and shear-thinning bioinks, which are hard to print using conventional microextrusion methods, are also easier to print using this method [19,20]. The screw-based method is widely used in the fused deposition melting (FDM) method of 3D printing, using polylactic acid (PLA), acrylonitrile-butadiene-styrene (ABS), etc. [21,22]. However, it is not widely used in biomedical applications due to the high stress exposure time from the syringe to the nozzle, the potential for contamination, and the high stress on the cells as the screw rotates to transport the bioinks, and it needs to be validated [23,24]. Furthermore, printing conditions include printing speed, dispenser feeding and pressure, nozzle shape and diameter, and chamber temperature [25,26], and these factors affect cell viability and printability [27,28]. In the screw-based method, factors that affect cell survival are quantitatively determined by measuring the shear stress on the cells [27,29], but measuring shear stress in combination with different printing conditions is difficult to achieve experimentally, and the ability to experimentally measure wall shear stress in each region of the bioprinting process is limited. On the other hand, computational fluid dynamics (CFD) analysis can predict the physical quantities and flow characteristics of all regions in the flow field. In particular, CFD analysis has the advantage of measuring the wall shear stress, which has a strong influence on the cell viability of the bioinks, in each region where the wall shear stress cannot be measured experimentally [30,31].

Bioinks used in bioprinting must exhibit three essential requirements. First, they must maintain printing temperatures that do not exceed physiological limits to prevent cell death [32,33]. Second, they should have optimal gelation conditions to maintain the structure after bioprinting [34,35]. Finally, they must have non-toxic properties that do not negatively affect the cells contained within the bioinks [36,37]. Bioink properties also include viscosity, viscoelasticity, hydrophilicity, and shear-thinning properties that affect printability and should be considered when selecting bioinks [38,39]. Currently, widely used biomaterials include hyaluronic acid, silk fibroin, calcium phosphate cement and cellulose [40,41,42,43]. Recently, Lee et al. [44] processed cellulose recovered from plum seeds and developed hybrid bioinks for 3D bioprinting to synthesize carboxymethyl cellulose (CMC) and combine it with alginate. However, determining the optimal printing conditions requires a lot of experience, time, and materials. Therefore, computational fluid dynamics (CFD) can be a suitable method for comparing and predicting the empirical and operational conditions of a 3D bioprinter and its environment. CFD analysis of bioinks with non-Newtonian fluids properties allows for the calculation of internal shear stresses and flow characteristics. Recently, Emmermacher et al. [45] used numerical simulations and analytical calculations to predict the mechanical stress on the cells, the pressure gradient, and the flow velocity. These predictions helped to optimize the printing process and to establish new bioink compositions. As follows, the development of an analysis system to predict the effect of bioinks on cell viability and functions by analyzing their physical properties could save time and money in bioink development [45].

**Figure 1:**
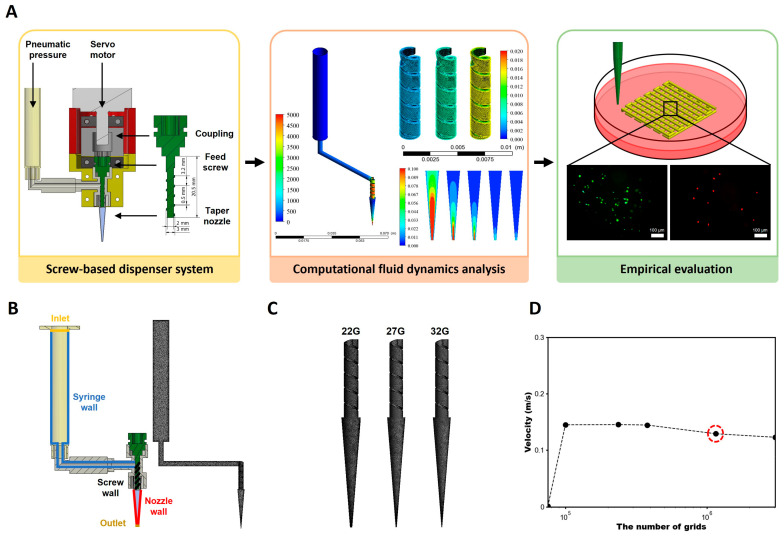
An analysis system for predicting bioink physical properties and cell viability from developed SDS. (**A**) Schematic diagram of this study consists of three steps. An SDS utilizing pneumatic pressure to transfer bioink and precisely extrude it using a feed screw was developed. Then, CFD analysis of the internal flow field was performed on an SDS. Empirical evaluation of cell viability was conducted using the developed SDS. Finally, the efficacy of the developed SDS was validated by a cell viability assay. (**B**) Classification of boundary with control volume and grid generation. The control volume is designed in the shape of the developed SDS, including syringe, screw, and nozzle. (**C**) Generated grids of screw and nozzle according to the different nozzle size. As nozzle number increases, the diameter of outlet decreases. The taper nozzles of 22, 27, and 32G (ID: 0.41, 0.21, and 0.10 mm, respectively) were used. (**D**) Evaluation of grid independence. The result of outlet velocity converged from about 1,156,645 grids. From the results of the grid independent test, the number of grids for CFD analysis was determined. SDS, screw-based dispenser system; CFD, computational fluid dynamics; ID, inside diameter.

In this study, we developed a screw-based dispenser system (SDS) (Figure 1A) suitable for high-viscous bioinks [46], and then developed an analysis system to predict the effect of the SDS on cell viability in bioinks through rheological measurement and CFD analyses. Then the bioprinting performance of the SDS was evaluated by comparing cell viability in high-viscous bioinks made from CMC and alginate. At first, the viscosity of bioinks was determined by rheological measurement, and the viscosity coefficient of correlation equations for CFD analysis was derived by non-linear regression analysis [47]. The CFD analysis was performed to visualize the internal flow field according to the printing conditions and to calculate velocity and wall shear stress in the SDS region as a function of nozzle size, feed screw rotational speed, and bioinks [48]. For validation purpose, the empirical mass flow rate was compared with the mass flow rate obtained from the CFD analysis by applying a safety factor. Finally, the biocompatibility of the developed SDS and carboxymethylcellulose/alginate (CMC/Al)-based high-viscous bioinks containing C2C12 cells was confirmed by assessing cell viability using a Live/Dead assay.

## 2. Materials and Methods

### 2.1. Materials for Bioprinting

Sodium carboxymethyl cellulose (CMC, 419281) was purchased from Sigma Aldrich (MO, St. Louis, USA). Dulbecco’s modified eagle’s medium (DMEM, LM 001-05), Dulbecco’s phosphate buffered saline (DPBS, LB 001-02), and Fetal bovine serum (FBS, S101-07) were purchased from WELGENE (Gyeongsan, Republic of Korea). Sodium alginate (Al, 7528-1405) was purchased from DAEJUNG (Siheung, Republic of Korea). Penicillin-streptomycin (PS, 15140122) was purchased from Gibco (Grand Island, NY, USA). Live/Dead assay kit (L3224), consisting of green-fluorescent calcein-AM and red-fluorescent ethidium homodimer-1, was purchased from Invitrogen (Waltham, MA, USA). C2C12 (CRL-1772) was purchased from ATCC (Manassas, VA, USA).

### 2.2. Configuration of Screw-Based Dispenser System

The SDS consisted of servo motor (AM8012, Beckhoff Automation, Verl, Germany), servo drive (AX5203, Beckhoff Automation, Verl, Germany), and coupling (SHR-29C, SUNGIL, Incheon, Republic of Korea), feed screw (DFS8-SS, VIEWEG, Kranzberg, Germany), air compressor (DC-661, KOLAVO, Seoul, Republic of Korea), dispenser, nozzle, etc. The feed screw had screw length of 20.5 mm, outside diameter of 3 mm, inside diameter (ID) of 2 mm, and distance per pitch of 3.2 mm. The type of nozzle was a taper nozzle, and its sizes were 22, 27, and 32G (ID: 420, 210, and 100 μm, respectively). The developed SDS used compressed air to transport the bioink and rotated the screw with the power of a servo motor to precisely extrude bioinks along the direction of nozzles.

### 2.3. Preparation of Bioinks

The CMC/Al bioinks were made of sodium carboxymethyl cellulose and sodium alginate. The experimental groups were composed of 3% (*w*/*v*) alginate with 10% (*w*/*v*) CMC (CMC10/Al3), 15% (*w*/*v*) CMC (CMC15/Al3), and 20% (*w*/*v*) CMC (CMC20/Al3), respectively. They were dissolved in a DMEM mixture containing 10% (*v*/*v*) FBS and 1% (*v*/*v*) PS, respectively, and stirred on a magnetic stirrer at 300 rpm for 2 h. An ultrasonic processor (VCX-750, SONICS, Newtown, CT, USA) generating 20 kHz ultrasound was used for 1 h to disperse inserts uniformly, and the resultant solutions were stored at 4 °C for 1 day for homogenization [49].

### 2.4. Rheological Characterization

The rheological properties of CMC/Al bioinks were measured using a modular rheometer (MCR-92, Anton Paar, Graz, Austria) equipped with a 25 mm diameter parallel plate (PP25, Anton Paar, Graz, Austria) and an adapter (C-PP50/XL, Anton Paar, Graz, Austria). One mL of each bioink was placed on the adapter maintained at 37 °C and equilibrated during measurements using the parallel plate. Viscosity measurements were performed at shear rates ranging from 0.01 to 100 s^−1^. The amplitude sweep test and frequency sweep test measured the storage modulus (G’) and loss modulus (G″) of the bioinks and provided information on the viscoelastic behavior, shear thinning, and subsequent recovery of the bioinks. In particular, the linear viscoelastic region (LVR) was obtained by an amplitude sweep test to measure shear strains ranging from 0.01 to 100% with an angular frequency of 1 rad/s. A frequency sweep test was evaluated at an angular frequency range of 0.1 to 100 rad/s with a shear strain of 1% where the LVR of each bioink intersected to confirm time-dependent fluid behavior.

### 2.5. Model Design for CFD

The geometry and grid for the CFD analysis were designed to encompass the control volume through which the flow passed in the actual geometry. The model included the syringe, screw, and nozzle of a developed SDS. Mesh generation was performed using ANSYS Meshing (v21, ANSYS, Canonsburg, PA, USA) with the maximum mesh size set for the entire shape. To perform CFD analysis based on nozzle size variations in the SDS, nozzle sizes of 22, 27 and 32G were included in the model (Figure 1C).

### 2.6. Governing Equations

CFD analysis of an SDS was performed based on calculations of steady-state, incompressible fluid. The mass conservation equation for the analysis is represented by Equation (1).

(1) $$\nabla \cdot \left(\rho \vec{U}\right)=0$$

Here, ρ represents density, and $\vec{U}$ denotes the velocity vector. The mass conservation equation is composed of the convective term on the left-hand side.

Furthermore, the momentum conservation equation is presented in Equation (2).

(2) $$\nabla \cdot \left(\rho \vec{U}\vec{U}\right)=-\nabla p+\nabla \cdot \vec{\tau }+\rho \vec{g}$$

Here, p is the pressure, $\vec{\tau }$ represents shear stress, and $\vec{g}$ indicates gravitational acceleration. The momentum conservation equation consists of the convective term on the left-hand side and pressure, stress, and gravity terms on the right-hand side. In CFD analysis, the mass conservation equation and momentum conservation equation are solved numerically to obtain velocity and pressure during the computational process. The CFD turbulence model for the stress term is applied to the K-Omega SST.

### 2.7. Analysis Conditions

To calculate the bioinks in the SDS, the bioinks were set as an incompressible fluid with fixed densities, where the densities were 1034.3 kg/m^3^ for CMC10/Al3, 1050.1 kg/m^3^ for CMC15/Al3 and 1064.9 kg/m^3^ for CMC20/Al3. The densities were measured on the basis of the mass corresponding to the volume of the bioinks actually produced. In addition, to represent the viscosity coefficient of bioinks in CFD analysis, a power-law model was used. The power-law model is shown in Equation (3) and is a general model for representing non-Newtonian fluids in terms of viscosity and shear rate.

(3) $$\mu =m{\dot{\gamma }}^{n-1}$$

where μ and $\dot{\gamma }$ are the viscosity coefficient and shear rate, respectively, and the parameters m and n are positive constants, called the consistency index and power law index, respectively. When n < 1, the fluid is called pseudoplastic, and when n > 1, it is called dilatant [50]. In addition, the viscosity coefficients of the bioinks in the power-law model used in this study are modified to be correlated.

For the CFD analysis, boundary conditions identical to the printing conditions conducted in the empirical evaluation of mass flow rate were set (Figure 1B). Pressure was set as the boundary condition at the inlet (0.1, 0.15, and 0.2 MPa). Non-slip conditions were set at the syringe wall and nozzle wall. To simulate the motor rotation in 3D bioprinting, feed screw rotational speeds of 60, 120, and 180 rpm were set as boundary conditions on the screw wall. Finally, the outlet was set the atmospheric pressure to dispense the bioinks.

In the numerical analysis, pressure and velocity were treated as coupled conditions, as mentioned above. For the spatial discretization, the gradient terms were treated using the Cell-Based Least Squares method, while the pressure term was treated using the Second Order method. The convective terms, the kinetic energy term, the dissipation rate term, and other related terms were all subjected to the Second Order Upwind scheme. In addition, the pseudo-transient technique was used to improve the stability of the solution in the steady-state analysis. In this study, the numerical analysis was conducted by ANSYS Fluent (v21, Canonsburg, PA, USA). Furthermore, these settings were applied as the basic numerical analysis configurations for the purpose of investigating the trends in velocity and wall shear stress with respect to geometry, bioinks and boundary conditions. To ensure convergence of the analysis, the residuals for the continuity and momentum equations were set to 1 × 10^−8^.

### 2.8. Empirical Evaluation of Mass Flow Rate

The empirical evaluation of the mass flow rate was to confirm the accuracy of the ejection repeatability of the developed SDS and to gain validation by comparing the mass flow rate with the CFD analysis. To empirically evaluate the mass flow rate, the mass flow rate was repeatedly measured five times per each experimental group for 1 min using a precision balance (WTC 200, RADWAG, Radom, Poland) with a readability of 0.001 g. Twenty seven different conditions according to changes in bioinks (CMC10/Al3, CMC15/Al3, and CMC20/Al3), nozzle size (22G, 27G, and 32G), and rotational speed of feed screw (60, 120, and 180 rpm) were analyzed. The pneumatic pressure, which is the minimum pressure required for bioinks transfer when the feed screw is not operating, was adjusted using an air compressor. Specifically, the pneumatic pressures of CMC10/Al3, CMC15/Al3, and CMC20/Al3 and were 0.05, 0.1, and 0.15 MPa, respectively.

### 2.9. Cell Viability Assay

To determine whether mechanical factors affect cell survival, we performed a cell viability assay using C2C12 cells from a mouse myoblast cell line [51]. The cells were cultured in 10 mL DMEM mixture containing 10% *(v/v)* FBS and 1% *(v/v)* PS. A CMC/Al bioink containing C2C12 cells at a concentration of 2 × 10^6^ cells/mL was fabricated, and the bioink was homogenized by carefully mixing with a spatula in a culture dish. Bioprinting of a 10 × 10 × 1 mm scaffold was performed in a culture dish. The control group was tissue culture polystyrene (TCP), in which cells were cultured in a culture dish, and the experimental groups were screw, in which bioprinting was performed using the developed SDS, and piston, in which bioprinting was performed using a piston-based dispenser system (PDS) commonly used in microextrusion methods [52]. The screw and piston experimental groups performed bioprinting using different bioinks (CMC10/Al3, CMC20/Al3) and nozzle sizes (22G, 27G, 32G). The printing speed was set to 300 mm/min, and the feed screw rotation speed was set to 120 rpm. The fabricated scaffolds were crosslinked with a 4% CaCl_2_ solution for mechanical stability. After crosslinking, the scaffolds were rinsed with DPBS, then removed by suction, and finally supplemented with DMEM in the culture dishes. A Live/Dead assay was performed 2 days after bioprinting to evaluate the cell viability caused by mechanical factors during the bioprinting process. For the Live/Dead assay, a staining solution was prepared by adding 5 μL calcein AM and 20 μL ethidium homodimer-1 to 10 mL DPBS. After removing the DMEM from the medium, 500 μL of the mixed staining solution was pipetted onto the scaffold, followed by incubation for 30 min. Live (green) and dead (red) cells were observed using the Leica K filter cube on a fluorescence microscope (DM3000, Leica, Wetzlar, Germany), and images were captured. Live and dead cells were counted, and live and dead cell images were merged using Image J software (v1.8.0, NIH, Bethesda, MD, USA). The cell viability was quantified by dividing the number of live cells by the total cell count, and the experiment was repeated five times.

### 2.10. Statistical Analysis

All data were presented as mean ± standard error, and one-way analysis of variance (ANOVA) and Duncan’s new multiple range test were performed at a significance level of *p* ≤ 0.05 to determine statistically significant differences in the mass flow test and the empirical evaluation of cell viability using R software (v4.3.2, R Foundation for Statistical Computing, Vienna, Austria).

## 3. Results and Discussion

### 3.1. Rheological Properties

**Figure 2:**
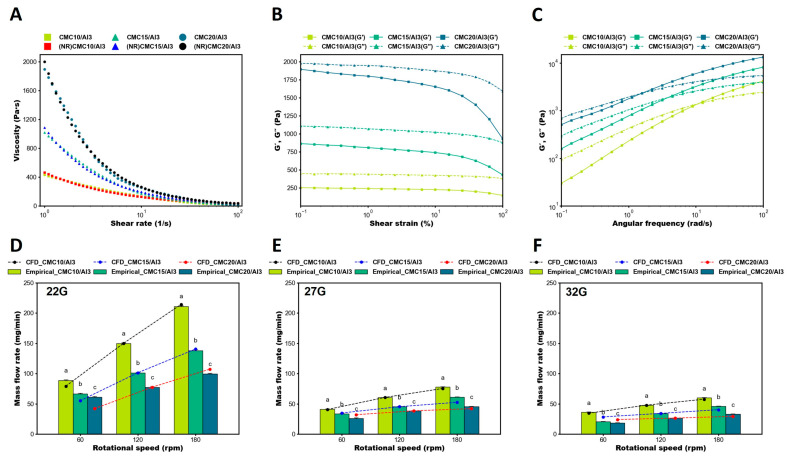
Rheological properties of CMC/Al bioinks and empirical evaluation of CFD analysis on mass flow rate. (**A**) Viscosity measurements based on shear rates to assess the behavior of bioinks. Non-linear regression was conducted to obtain non-Newtonian fluid viscosity for CFD analysis, and the results were similar to the experimental results. (**B**) Amplitude sweep test based on shear strain to assess the behavior. LVR was confirmed for the determination of shear strain. (**C**) Frequency sweep test based on angular frequency to verify the time-dependent behavior. The bioinks were in a sol state at low frequencies and transited to a gel state at high frequencies. (**D**–**F**) Comparison of empirical evaluation with CFD analysis of mass flow rate using 22 (**D**), 27 (**E**), and 32G (**F**) taper nozzle. An experimental correction factor was multiplied for the mass flow CFD analysis results, and the slope tendencies of the mass flow CFD analysis and empirical results were compared. Error bars indicate the standard error of the mean. Same letter indicates statistical insignificance (Duncan’s new multiple range test, *p* ≤ 0.05). LVR, linear viscoelastic region.

Shear thinning, a characteristic of non-Newtonian fluids, is a physical property of the fluid and is a phenomenon in which the viscosity of the fluid decreases as the shear stress increases. Appropriate viscosity and flow behavior in bioinks have an advantage to maintain their shape without causing stress on the cells during bioprinting. To confirm this, the viscosity of the CMC/Al bioinks was measured using a rheometer (Figure 2). It was observed that the viscosity increased as the CMC content increased, and all types of CMC/Al bioinks exhibited shear thinning characteristics. In particular, CMC20/Al3 exhibited high viscosity, reaching over 2000 Pa·s (Figure 2A). An amplitude sweep test was performed to confirm the deformation behavior and the upper limit at which the structure of the bioink would not break. The CMC/Al bioinks showed LVR at a shear strain rate of 0.1 to 1%, and the upper limit at which the structure did not break decreased with increasing CMC content (Figure 2B). In addition, frequency sweeps were performed to verify the time-dependent behavior of the bioinks. It was observed that as the CMC content increased, the storage modulus (G′) became higher than the loss modulus (G″) even at low speeds, representing the CMC content in bioinks was an important factor of gel state (Figure 2C). The results confirmed that CMC/Al bioinks were suitable as bioinks due to their shear thinning properties, minimizing the shear stress that affected the cells during bioprinting. To apply the previously obtained viscosity coefficient of CMC/Al bioinks to CFD analysis, a non-linear regression analysis was performed, where the viscosity coefficient was the result of shear rates from 1 to 100 1/s in Equations (4)–(6).

(4) $$\mu =463.38{\dot{\gamma }}^{0.43417-1}\;(CMC10/Al)$$

(5) $$\mu =1087{\dot{\gamma }}^{0.25397-1}\;(CMC15/Al)$$

(6) $$\mu =1999.3{\dot{\gamma }}^{0.11644-1}\;(CMC20/Al)$$

Here, the coefficients of determination of the non-linear regression analysis results are 0.985, 0.993, and 0.996, respectively, and the RMS errors are 15.9, 24.5, and 33.7, respectively (Figure 2A). The coefficients of determination and RMS error of the non-linear regression resulted in a regression equation that is very closely related to the true fit.

### 3.2. Comparison of Empirical Evaluation and CFD Analysis in Mass Flow Rate

In bioprinting, the accuracy of ejection repeatability is critical for printing precise structures such as biological tissues and organs, and establishing printing conditions can help ensure consistent printing results. Therefore, the empirical evaluation of mass flow rate can confirm the accuracy of the ejection repeatability. In addition, the results of the mass flow empirical evaluation were compared to confirm the reliability of the CFD analysis results. To evaluate the validity of the grids generated for CFD analysis, a grid independence test was performed for a nozzle geometry with a mid-range size of 27G (Figure 1D). The grid independence test for grid size optimization was performed ranging from 75,208 to 3,034,394 grids and was evaluated based on the convergence of the average velocity at the nozzle outlet. The test results indicated that the outlet velocity converged within approximately 4.7% for 1,156,645 grids. Consequently, the CFD analysis was carried out using 1,156,645 grids with grid sizes within 0.25 mm.

As a result of empirical evaluation of mass flow, it was observed that the mass flow rate was inversely proportional to the nozzle size, the feed screw rotational speed, and the CMC content in the bioink (Figure 2). It was confirmed that precise ejection was achieved even with highly viscous CMC20/Al3 by rotating the feed screw to transport the material uniformly, suggesting that the developed SDS was capable of high-precision bioprinting with high-viscous materials (Figure 2D–F). The mass flow rate from the CFD analysis was calculated by multiplying the average velocity at the outlet of the nozzle, the density of the bioink, and the cross-sectional area of the outlet. A safety factor was applied to the CFD results to account for the error in the mass flow rate at 120 rpm, and a value of 0.15, 0.18, and 0.53 was applied to the CFD results, depending on the size of the nozzle, by performing an average calculation for the bioink. Furthermore, to verify the validity of the CFD simulation, a comparison was made between the empirical evaluation and the CFD simulation of the mass flow rate. Since the CFD simulation did not take into account the SDS such as errors of printing conditions, losses in the machine, and ambient environmental conditions, which might lead to errors in the empirical evaluation, an experimental correction factor [53] was used to adjust the CFD simulation results. The experimental correction factor was applied at a screw rotational speed of 120 rpm, which was the median speed for each bioink, to match the empirical evaluation and CFD simulation data, and the results were evaluated by comparing the trend of mass flow rate with speed for each bioink. In the CFD analysis, the ejected mass flow rate was calculated by the velocity of the nozzle outlet and the density of the bioink. The results were compared with the empirical evaluation using an experimental correction factor. Although small differences were observed for high-viscous bioinks, the general trend was consistent. The results demonstrate that the CFD simulation of CMC/Al bioinks in SDS was well verified.

### 3.3. Flow Field of CFD Analysis

**Figure 3:**
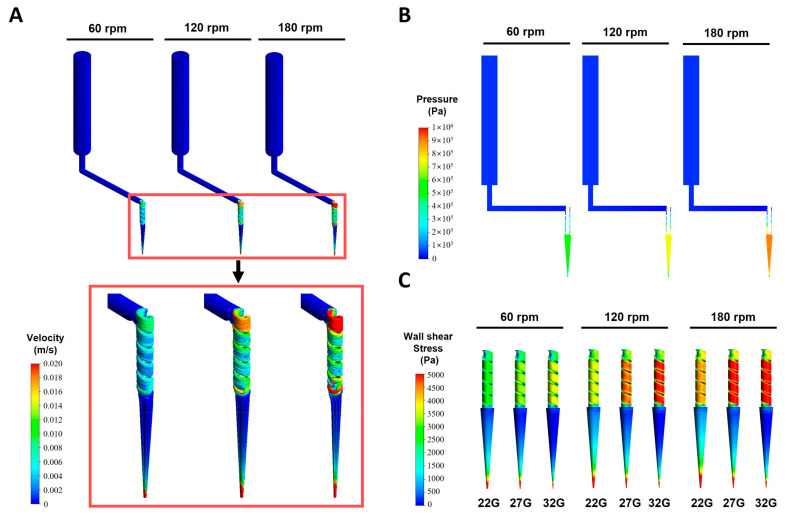
CFD analysis of SDS. (**A**) Representative streamlines at 27G and CMC10/Al3 with different rotational speeds. The results indicated that the velocity in the screw region increased as rotational speed increased. Also, the increase in velocity distribution at the nozzle confirmed the increase in mass flow rate. (**B**) Pressure distribution at 27G and CMC10/Al3 with different rotational speeds. In the screw and nozzle area, high pressure was generated, and the pressure in the nozzle increased as the rotational speed increased. (**C**) Wall shear stress distribution with nozzle size and rotational speed at screw and nozzle. Due to the boundary condition of rotational speed, higher wall shear stresses were generated in the screw region than those in the nozzle region. The wall shear stress increased as the nozzle size decreased and was proportional to the rotational speed.

The CFD analysis is a very suitable method to calculate the velocity, internal pressure, and wall shear stress in bioprinting. The CFD analysis provided the velocity, internal pressure, wall shear stress of the internal flow field with the CMC/Al bioinks and nozzle sizes (Figure 3). The velocity distributions and streamlines of the CMC15/Al bioink and a nozzle size of 27G were presented (Figure 3A). Here, the internal velocity distribution of the bioinks was derived to obtain consistent results with respect to the boundary conditions used in the simulation, and, in the case of the streamlines, to represent the continuous flow of fluids during the bioprinting process. The results showed that the highest velocities as a function of rotational speeds occurred in the screw and nozzle regions, with relatively low velocities in the syringe region. On the other hand, looking at the velocity distribution at the nozzle outlet, the velocity increased with rotational speed, with the largest velocity occurring at 27G (Figure S1). In general, for the same density in the internal flow, the outlet velocity should decrease as the cross-sectional area of the outlet decreases under the same boundary conditions. However, when the rotational velocity is set as the boundary condition, the results may not be consistent with the cross-sectional size due to the non-linear viscous effect, such as in the case of the SDS [54]. In addition, when converting this to mass flow rate, the mass flow rate decreased as the nozzle size decreased, as shown in the previous results in Section 3.1. In the case of the nozzle’s internal velocity distribution, as the rotation speed increased and the nozzle size increased, a larger velocity distribution occurred (Figure S2). This is owing to the fact that the boundary condition of rotational speed does not affect the syringe region. For the internal pressure, the screw and nozzle pressures increased in proportion to the rotational speed, similar to the results for velocity (Figure 3B). On the other hand, as the nozzle size decreased, the wall shear stress increased in the screw region but decreased in the nozzle region (Figure 3C). As the bioink content increased, the wall shear stress increased (Figure S3). Also, as the rotational speed increased, the wall shear stress increased in both the screw and nozzle regions.

**Figure 4:**
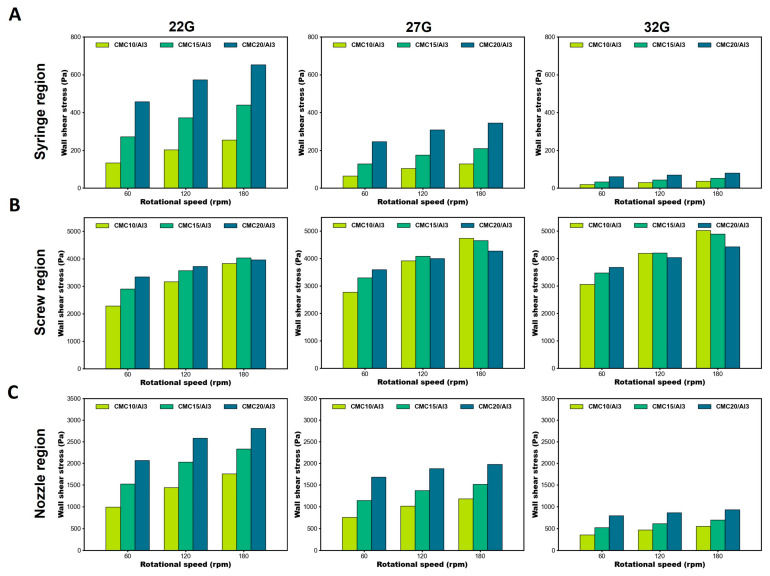
Wall shear stress with different rotational speeds and bioinks. (**A**) Wall shear stress of syringe region. The wall shear stress decreased as the nozzle size decreased. When the rotational speeds and bioinks content increased, the wall shear stress proportionally increased. (**B**) Wall shear stress of screw region. The wall shear stress increased as the nozzle size decreased. At rotational speeds of 120 and 180 rpm, the wall shear stresses became non-linear. (**C**) Wall shear stress of nozzle region. Similar to the area of the syringe, the wall shear stress was inversely proportional to a decreasing nozzle size and proportional to rotational speed, bioink content, and shear stress.

Regarding the variation in the wall shear stress in each bioprinting region (Figure 4), in the syringe region, the wall shear stress decreased as the nozzle size decreased and increased as the rotational speed and bioink content increased (Figure 4A). In the screw region, the wall shear stress increased as the nozzle size decreased (Figure 4B). On the other hand, the wall shear stress increased as the bioink content increased at 60 rpm, while the results were non-linear at 120 and 180 rpm. These results are attributed to the shear thinning of the non-Newtonian fluid, which was directly affected by the high rotational speeds experienced in the screw region. In addition, non-Newtonian fluids exhibit sensitive changes in shear stress and other variables with rotational speed and rheological properties [55]. The wall shear stress trends in the nozzle region were similar to those in the syringe region. As the nozzle size decreased, the wall shear stress decreased, and as the rotational speed and bioink increased, the wall shear stress increased (Figure 4C). Based on the results, it was confirmed that the largest wall shear stresses occurred in the screw and nozzle regions during the printing process in the SDS, with a nonlinear effect of rotational speed, especially in the screw region. It suggests that the development and analysis of the screw region has a significant impact on cell survival in bioinks. In addition, cell viability can be inferred by examining the literature on cell viability and comparing it to the CFD simulation results.

### 3.4. Cell Viability

**Figure 5:**
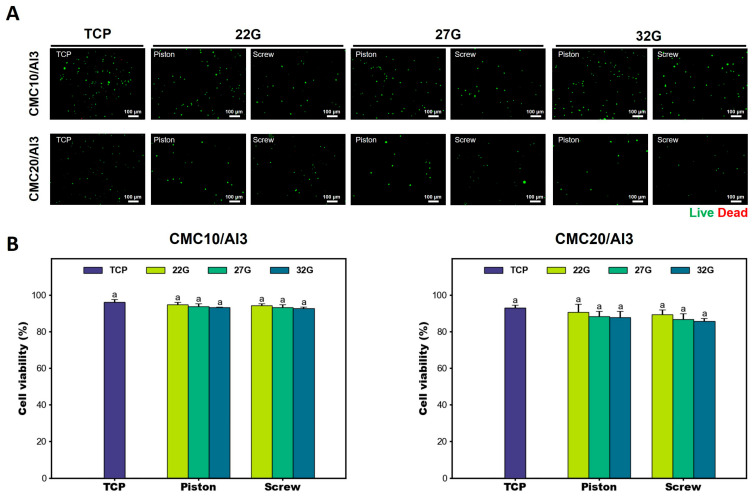
Cell viability test to evaluate the validation of SDS. (**A**) Empirical evaluation of C2C12 cell viability in the 3D-printed structures using a Live/Dead assay. Live/Dead analysis was performed 2 days after bioprinting. (**B**) Quantitative analysis results of cell viability of CMC/Al bioinks according to bioprinting methods. Percentages were determined by dividing the number of viable cells by the total number of cells. Image J software (v1.8.0) was used to count live and dead cells in the acquired images by Live/Dead analysis. There was no statistical significance in cell viability between bioprinting with the PDS and bioprinting with the SDS. Error bars indicate the standard error of the mean. The same letter indicates statistical insignificance (Duncan’s new multiple range test, *p* ≤ 0.05). PDS, piston-based dispenser system.

The developed SDS pneumatically transported bioink-containing cells, which were precisely dispensed through a nozzle by rotating the feed screw. Cells were affected by shear stress during the bioprinting process due to mechanical factors such as pneumatic pressure, screw, and nozzle size. Therefore, to determine whether mechanical factors affect cell viability, we assessed cell viability using the Live/Dead assay 2 days after bioprinting. The CMC/Al bioinks were cross-linked using CaCl_2_, and live and dead cells were observed within the structures. The cell viability of bioprinting with the PDS and SDS were slightly lower than that of the TCP group, but the difference was not significant. It was also found that there was a non-significant difference between the cell viability of bioprinting with the PDS and the cell viability of the developed SDS (Figure 5). Lemarié, L. et al. [54] showed that wall shear stresses of 4789 to 5687 Pa were generated when using a 0.2 mm conical nozzle, and cell viability was 77.2 to 91.4%. The results of CFD analysis in this paper showed that wall shear stresses of 2779~4278 Pa were generated at 27G (0.2 mm) and a cell viability of 86.7~93.8%. Therefore, this means that the mechanical factors of the developed SDS generate wall shear stress on the cells embedded in the bioink, but do not significantly affect cell viability. The cell viability results demonstrated that the developed SDS is possible for bioprinting, and that bioprinting with high-viscous bioinks is feasible. It is also expected that the developed SDS will be able to enter the food field, where printing with high-viscous food materials can be used to promote innovative designs and characteristics of food products to provide consumers with a healthy life [56,57].

The field of bioprinting refers to the technology of printing biological tissues and organs, which requires a combination of biomaterials, cell supply and viability, bioprinter types, biological compatibility, and functionality [58,59]. In addition, to scale up to larger tissue structures, three-dimensional structures must be maintained after printing, and they must be printed precisely [60]. Quantitative discharge and high-precision printing are required to stabilize the structure and achieve precision printing, and the development of a high-precision 3D bioprinter with the developed SDS is expected to contribute to the possibility of bioprinting large tissue structures. Furthermore, the CFD analysis of the developed SDS can be used to analyze and evaluate any biomaterial using multiphase flow, composite material flow, etc., which have been difficult to analyze until now, and can predict printing conditions and cell viability in advance. This means that the time, material, and energy consumption of experiments such as setting up printing conditions and cell viability are reduced, and this is highly effective.

## 4. Conclusions

In this study, we developed SDS suitable for high-viscous bioinks and a tool to predict its impact on cell viability in bioinks through rheological and CFD analyses. CFD analysis showed that the syringe and nozzle regions exhibited consistent velocity distributions and wall shear stresses with nozzle sizes, bioink contents, and rotational speeds. On the other hand, the screw region exhibited the highest wall shear stresses and non-linear results with rotational speed, suggesting that further screw region development and multivariate studies are needed. In the cell viability evaluation, a non-significant difference was found in the Live/Dead assay after bioprinting compared to the piston method, which suggests that the developed SDS is suitable for bioprinting. Furthermore, the developed bioink analysis tool is applicable to a wide range of systems and materials, and the stresses applied to the bioinks can be analyzed to predict the effect on cell survival when the bioinks containing cells are ejected, saving time and money in bioink development.

## Data Availability

Data available on request due to restrictions privacy or ethical. The data presented in this study are available on request from the corresponding author.

## Supplementary Materials

The following supporting information can be downloaded at: https://www.mdpi.com/article/10.3390/polym16081137/s1, Figure S1: Velocity distributions and profile at outlet. As the rotational speeds increased, the outlet velocity is increased. Additionally, when looking at the velocity distribution according to nozzle size, the highest velocity occurred at 27G, followed by 32G and 22G; Figure S2: Velocity distributions of nozzle cross-section. As the rotational speed increased, the velocity distribution in the nozzle increased, and as the nozzle size decreased, the velocity distribution also decreased. On the other hand, no significant variations in velocity distribution occurred at the nozzle for the bioink content; Figure S3: Wall shear stress distribution at screw and nozzle. Figure 3C additional supplementary data.
