# Supplementary material for: Computational Fluid Dynamics Analysis and Empirical Evaluation of Carboxymethylcellulose/Alginate 3D Bioprinting Inks for Screw-Based Microextrusion
Source: Polymers (Basel). 2024 Apr 18;16(8):1137. doi: 10.3390/polym16081137 (PMC11054610; doi:10.3390/polym16081137)
Supplement: Supplementary file 1 [file polymers-16-01137-s001.zip › polymers-2934844-supplementary.pdf]

Supplementary File

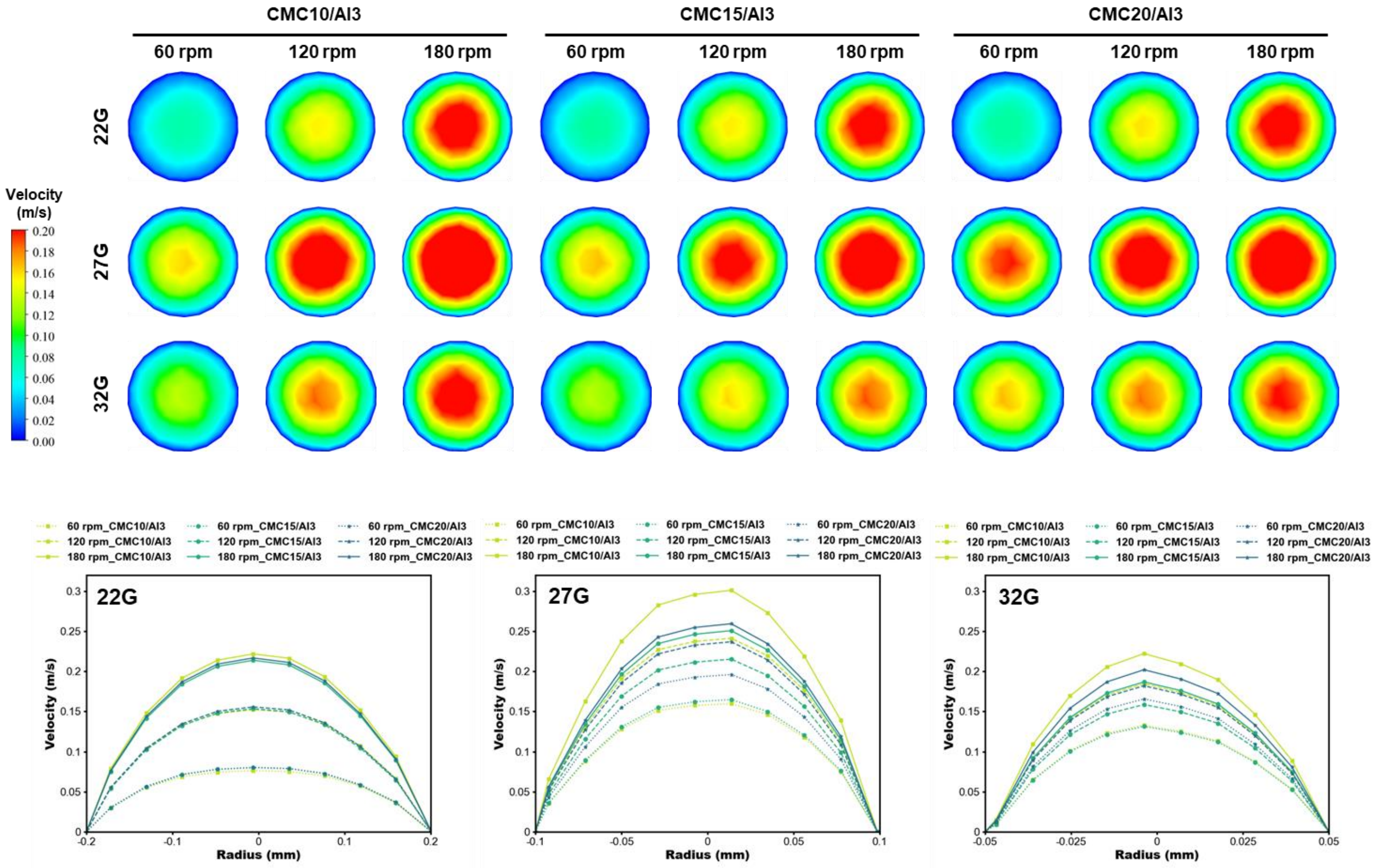

Figure S1. Velocity distributions and profile at outlet. As the rotational speeds increased, the outlet velocity is increased. Additionally, when looking at the velocity distribution according to nozzle size, the highest velocity occurred at 27G, followed by 32G and 22G.

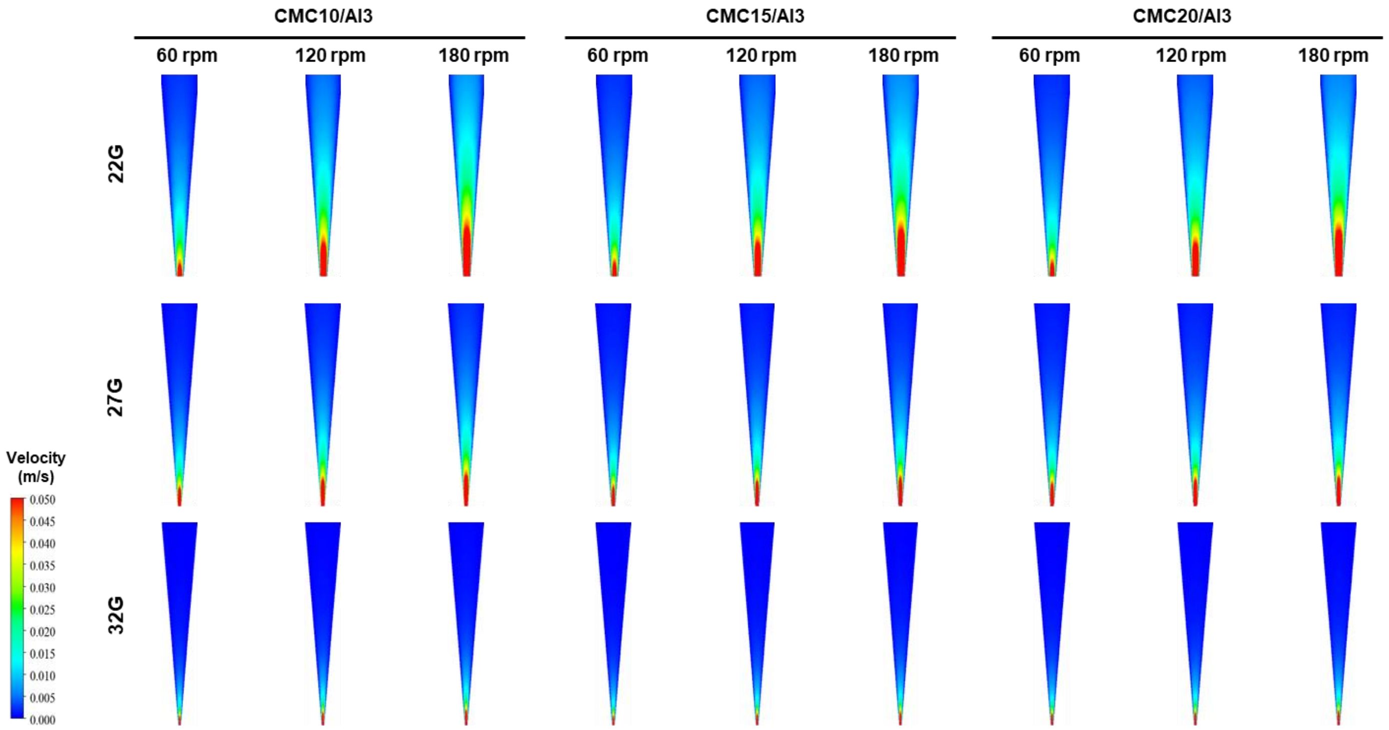

Figure S2. Velocity distributions of nozzle cross-section. As the rotational speed increased, the velocity distribution in the nozzle increased, and as the nozzle size decreased, the velocity distribution also decreased. On the other hand, no significant variations in velocity distribution occurred at the nozzle for the bioink content.

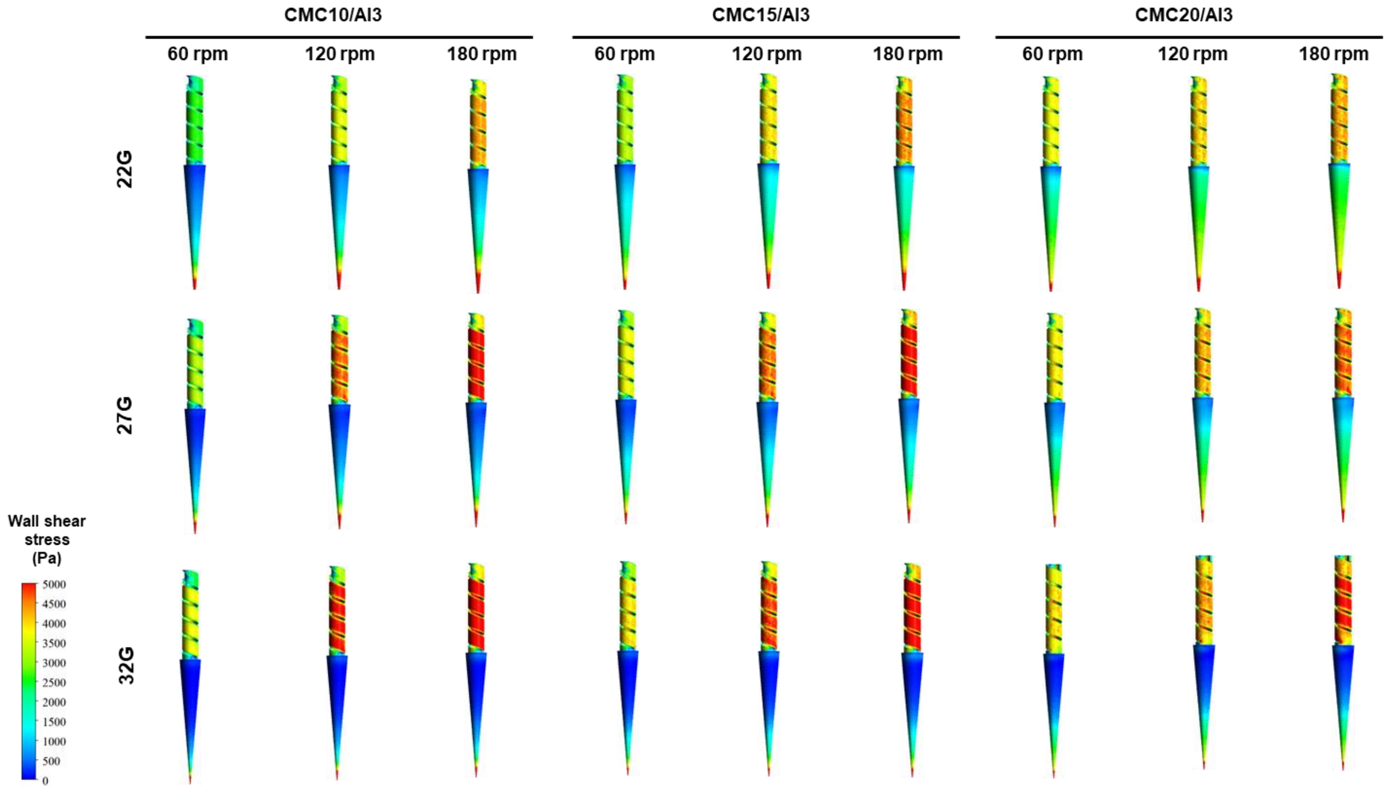

Figure S3. Wall shear stress distribution at screw and nozzle. Figure 3C additional supplementary data.
